# Skin-Whitening Effects of Cannabinol (CBN) Through Melanin Inhibition in B16F10 Melanoma Cells

**DOI:** 10.3390/ijms262110752

**Published:** 2025-11-05

**Authors:** Joon-Hee Han, Jong-Hui Kim, Min Hong, Byeong-Ryeol Ryu, Jung Dae Lim, Keun-Cheol Kim, Tae-Hyung Kwon

**Affiliations:** 1 Institute Biological Resources, Chuncheon Bioindustry Foundation, Chuncheon 24232, Republic of Korea; cbfhjh@cbf.or.kr (J.-H.H.); jonghe5820@cbf.or.kr (J.-H.K.); fabre_min@cbf.or.kr (M.H.); 2 Institute of Cannabis Research, Colorado State University—Pueblo, Pueblo, CO 81001, USA; byeongryeol.ryu@csupueblo.edu; 3 Department of Bio-Health Convergence, Kangwon National University, Chuncheon 24341, Republic of Korea; ijdae@kangwon.ac.kr; 4 Department of Biological Sciences, Kangwon National University, Chuncheon 24341, Republic of Korea; kckim@kangwon.ac.kr

**Keywords:** skin-whitening, melanogenesis, cannabinol, B16F10 melanoma cells, tyrosinase, melanin

## Abstract

Melanogenesis, the key biological process underlying skin hyperpigmentation, is tightly regulated by complex molecular signaling pathways. Consequently, targeting molecular regulators of this pathway is a crucial strategy for developing effective skin-whitening agents. Cannabinol (CBN), a minor cannabinoid, has been largely unexplored owing to its role in modulating skin pigmentation. This study aimed to elucidate the molecular mechanisms of CBN’s depigmenting effects using an α-MSH-induced B16F10 melanoma cell model. High-purity CBN was obtained via conversion of cannabidiol (CBD) and confirmed by HPLC. CBN significantly inhibited melanin synthesis and tyrosinase activity in a concentration-dependent manner, without any cytotoxicity. Furthermore, we investigated CBN’s impact on the melanogenesis signaling cascade. Our analysis revealed that CBN significantly downregulated the mRNA and protein levels of key melanogenic master regulators, including MITF, TYR, TYRP1, and TYRP2. Importantly, we also observed that CBN treatment selectively suppressed the protein phosphorylation of upstream signaling molecules such as p38 and JNK MAP kinases and NF-κB, while ERK phosphorylation remained unaffected. This finding indicates that its mechanism of action involves the selective modulation of pro-melanogenic signaling components. Collectively, these findings demonstrate that CBN effectively modulates the melanogenesis signaling pathway by targeting both upstream kinases and downstream melanogenic genes. These findings suggest that CBN holds great promise as a bioactive agent for skin-whitening applications and warrants further research to confirm its clinical efficacy and safety.

## 1. Introduction

In recent years, increasing interest in skin brightening and tone correction has positioned skin pigmentation control as a key area of research in both cosmetic and biomedical industries [1]. Consequently, the global skincare market has witnessed a significant surge in products boasting whitening functionalities, directly propelled by the increasing consumer focus on mitigating signs of aging and treating hyperpigmentation [2,3]. Particularly in Asian countries, where even and bright skin tones are highly preferred, the development of ingredients that inhibit melanin synthesis has become a central focus of functional cosmetics research [1,4]. Furthermore, skin whitening is not only pursued for aesthetic purposes but also contributes to photoprotection, inflammation reduction, and the prevention of pigmentation-related skin disorders, highlighting its importance in both therapeutic and preventive dermatology [5,6,7,8].

Melanin, a phenolic polymer synthesized by melanocytes in the epidermis, is the principal determinant of human skin color, overshadowing other pigments such as hemoglobin and β-carotene due to its concentration and distribution. The two primary types of melanin are eumelanin, a black-to-brown insoluble pigment, and pheomelanin, a red-yellow soluble pigment [9]. Melanogenesis is a complex biosynthetic pathway initiated by ultraviolet (UV) radiation, which activates tyrosinase (TYR), the rate-limiting enzyme that catalyzes the oxidation of L-tyrosine to L-DOPA and subsequently to DOPA quinone. This pathway is further modulated by tyrosinase-related proteins (TYRP1 and TYRP2) and microphthalmia-associated transcription factor (MITF), which collectively regulate melanocyte function and melanin production [9,10]. In addition to these core enzymes and transcription factors, melanogenesis is controlled by upstream signaling cascades, including the mitogen-activated protein kinase (MAPK) pathway [11]. This pathway is composed of key kinases, such as extracellular signal-regulated kinase (ERK), c-Jun N-terminal kinase (JNK), and p38, and plays a crucial role in regulating MITF activation and subsequent melanin production [12]. Specifically, the activation of the ERK and JNK pathways is known to suppress MITF, leading to reduced tyrosinase expression and melanin synthesis. In contrast, the p38 pathway can both positively and negatively regulate melanogenesis depending on the context. Therefore, targeting these upstream signaling molecules is a promising strategy for controlling hyperpigmentation at the fundamental level.

While melanin provides crucial photoprotection by absorbing UV radiation and scavenging reactive oxygen species, its overproduction, often induced by chronic UV exposure, contributes to pigmentary disorders, such as melasma, lentigines, and photoaging [5,6]. Therefore, tyrosinase inhibition is a pivotal strategy for the development of cosmetic and therapeutic agents aimed at controlling hyperpigmentation. Kojic acid and arbutin inhibit tyrosinase activity. Specifically, kojic acid acts through copper ion chelation, whereas arbutin acts via competitive substrate inhibition. However, the clinical use of these agents is limited by adverse effects, such as skin irritation and cytotoxicity [13]. Thus, there is an urgent need for novel, safe, and efficacious natural compounds that can modulate melanogenesis with minimal side effects.

Cannabinol (CBN) is a mildly psychoactive cannabinoid, naturally formed as a degradation product of tetrahydrocannabinol (THC) via oxidation and aging [14]. Although THC is typically found in trace amounts in fresh *Cannabis sativa* plants, its concentration increases when it undergoes thermal and oxidative decomposition. In addition to this natural pathway, CBN can be synthesized from cannabidiol (CBD) through chemical processes, offering an alternative production route. Unlike THC, CBN exhibits a significantly lower affinity for cannabinoid receptors CB1 and CB2, and is thus considered to have minimal psychoactive effects [14]. Recent studies have focused on the therapeutic potential of CBN, including its anti-inflammatory, analgesic, neuroprotective, and antibacterial properties [15,16]. In particular, CBN has been reported to exhibit antioxidant activity, which suggests potential applications in dermatology and skin aging [4,9]. As oxidative stress and inflammation stimulate melanogenesis via tyrosinase activation and MITF signaling, compounds with antioxidant capacities may serve as effective agents for modulating melanin synthesis [5,9].

In this study, we investigated the potential skin-whitening effects of cannabinol, a minor cannabinoid obtained through iodine-mediated conversion and CPC purification from cannabidiol. Using the B16F10 melanoma cell line, a widely used in vitro model for studying melanogenesis, we evaluated the impact of CBN on melanin production and tyrosinase activity [2,4]. We assessed whether CBN could suppress melanin accumulation and enhance the inhibition of tyrosinase activity in cells stimulated with α-melanocyte stimulating hormone (α-MSH). Specifically, we analyzed changes in the expression of key melanogenic markers, including TYRP1, TYRP2, TYR, and MITF, at both mRNA and protein levels. Through these investigations, we aimed to demonstrate the potential of CBN as a novel bioactive compound for skin whitening.

## 2. Results

### 2.1. Isolation and Characterization of Purified CBN

**Figure 1:**
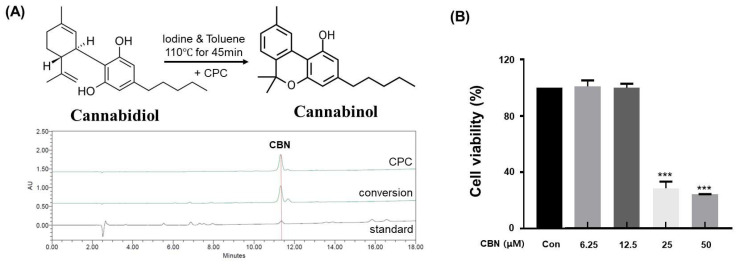
Analysis of CBN conversion and purification, and its subsequent cytotoxicity in B16F10 cells. (**A**) High-performance liquid chromatography (HPLC) chromatograms of standard CBN, the CBN conversion product from cannabidiol (CBD), and the final purified CBN. The top chromatogram shows the high purity of the CBN obtained via centrifugal partition chromatography (CPC). The middle chromatogram displays the raw conversion product, while the bottom chromatogram of the standard CBN confirms the identity of the compound. (**B**) Cytotoxicity of CBN on B16F10 melanoma cells. The cells were treated with various concentrations of CBN for 48 h, and cell viability was measured using a CCK-8 assay. The graph demonstrates that CBN exhibited no significant cytotoxicity up to a concentration of 12.5 μM. Results are presented as the relative percentage of the untreated control group and expressed as the mean ± standard deviation (*n* = 3 per group). *** *p* < 0.001.

In this study, cannabidiol (CBD) extracted from *Cannabis sativa* L. was successfully converted to cannabinol (CBN) via iodine-mediated oxidative cyclization under reflux in toluene. This metal-free conversion method offers several advantages over conventional acid- or metal-catalyzed protocols, including reduced toxicity, minimal by-product formation, and compatibility with downstream purification processes. Following the reaction, the mixture was subjected to centrifugal partition chromatography (CPC), a liquid–liquid separation technique that does not require solid stationary phases. The conversion efficiency of CBD to CBN under the iodine-mediated, metal-free oxidative cyclization was calculated to be 87.6 ± 2.3%, as determined by HPLC quantification of the reaction mixture. The employed biphasic solvent system (n-hexane:ethyl acetate:methanol:water, 5:5:5:5, *v*/*v*/*v*/*v*) provided excellent phase separation and resolution, with the organic phase serving as the stationary phase and the aqueous phase as the mobile phase in descending mode (Table 1). The operating conditions were optimized at a flow rate of 10 mL/min and a rotation speed of 1600 rpm. High-performance liquid chromatography (HPLC) of the collected fractions confirmed successful enrichment and isolation of CBN. The final purified product exhibited a purity of 99.0% (Figure 1A), significantly exceeding the typical purity range (85–95%) reported for conventional purification approaches. Additionally, the crude CBN obtained before CPC purification showed an HPLC purity of 86.23%, whereas the highly purified CBN after CPC exhibited a purity of 99.23%. This comparison clearly demonstrates that the CPC purification process effectively removed impurities and enhanced the overall purity of CBN, supporting its superior quality and reliability for subsequent bioactivity assays.

### 2.2. Assessment of B16F10 Cell Viability

To evaluate the cytotoxic effects of cannabinol (CBN) on B16F10 melanoma cells, a CCK-8 assay was conducted. Untreated cells served as the control group, while CBN was administered at concentrations of 6.25, 12.5, 25, and 50 μM. Significant reductions in cell viability were observed at concentrations equal to or exceeding 25 μM. However, no statistically significant differences in viability were detected at concentrations at or below 12.5 μM compared to the control (Figure 1B). Based on these findings, concentrations of 2.5, 5, and 10 μM, which did not elicit cytotoxic effects, were non-cytotoxic and thus suitable for evaluating CBN’s anti-melanogenic effects.

### 2.3. Assessment of Tyrosinase Inhibitory Activity

**Figure 2:**
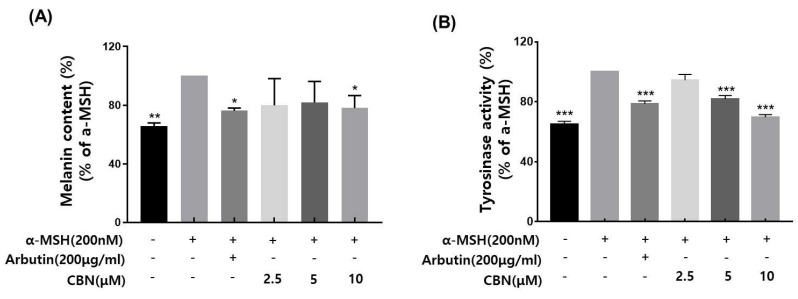
Effects of CBN on α-MSH-induced melanogenesis in B16F10 melanoma cells. (**A**) Melanin content was measured in B16F10 melanoma cells stimulated with α-melanocyte-stimulating hormone (α-MSH, 200 nM) to induce melanogenesis and treated with various concentrations of cannabinol (CBN 2.5, 5, and 10 µM) for 48 h. Arbutin (200 µg/mL) was used as a positive control. (**B**) Tyrosinase activity was evaluated under the same treatment conditions. Cell lysates were incubated with L-DOPA as a substrate, and the dopachrome formation was measured spectrophotometrically. Data are presented as the mean ± standard deviation (*n* = 3 per group), relative to the α-MSH-treated control. Statistical significance was determined using one-way ANOVA followed by post hoc analysis, as described in the Materials and Methods Section. * *p* < 0.05, ** *p* < 0.01, *** *p* < 0.001.

Tyrosinase is a key rate-limiting enzyme in melanogenesis, catalyzing the oxidation of the amino acid L-tyrosine to L-DOPA and subsequently to dopaquinone, which leads to melanin synthesis. As a critical regulatory enzyme in the pigmentation pathway, tyrosinase is the primary target of skin-whitening agents. Various natural and synthetic inhibitors, including arbutin, kojic acid, licorice extract, and mulberry extract, have been developed and widely employed in cosmetic formulations to suppress melanin production [17,18]. In the present study, we evaluated the tyrosinase inhibitory potential of cannabinol (CBN), using arbutin as a positive control. CBN was tested at concentrations of 2.5, 5, and 10 μM. CBN suppressed the tyrosinase activity in a concentration-dependent manner (Figure 2A). Notably, CBN exhibited tyrosinase inhibition rates of 94.4 ± 3.9%, 81.8 ± 2.4% and 69.7 ± 1.7% at 2.5, 5, and 10 μM, respectively, indicating a pronounced skin-whitening efficacy.

### 2.4. Melanin Content Reduction by CBN

We evaluated the inhibitory effects of cannabinol (CBN) on melanin biosynthesis in α-melanocyte-stimulating hormone (α-MSH)-stimulated B16F10 melanoma cells. Based on prior cytotoxicity results, non-cytotoxic concentrations of CBN (2.5, 5, and 10 μM) were selected for this assay. Following α-MSH induction, which upregulates melanogenesis via the cAMP/MITF signaling axis, CBN treatment resulted in a concentration-dependent re-duction in intracellular melanin levels [9]. At the lowest concentration of 2.5 μM, CBN exhibited a melanin inhibition rate of average 79.9% ± across three independent measurements, indicating a strong suppressive effect on melanin production (Figure 2B).

### 2.5. qRT-PCR Validation of Melanogenic Gene Expression

**Figure 3:**
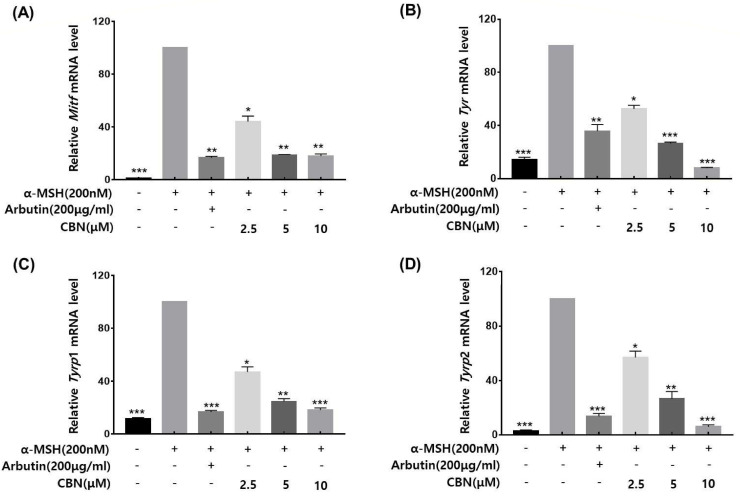
CBN downregulates the mRNA expression of key melanogenic genes. The anti-melanogenic effects of CBN were validated at the gene expression level by quantitative real-time PCR (qRT-PCR). B16F10 melanoma cells were treated with various concentrations of CBN for 48 h. The analysis demonstrated that CBN treatment resulted in a dose-dependent downregulation of key melanogenic genes and their master transcription factor, specifically (**A**) *Mitf*, (**B**) *Tyr*, (**C**) *Tyrp*1, and (**D**) *Tyrp*2. The graphs show the relative mRNA expression levels of key melanogenic genes, normalized to GAPDH and expressed as a percentage of the untreated control. All data are presented as the mean ± SD from three independent experiments. * *p* < 0.05, ** *p* < 0.01, *** *p* < 0.001.

To validate whether the anti-melanogenic effects of CBN were regulated at the gene expression level, we performed quantitative real-time PCR (qRT-PCR) on B16F10 melanoma cells treated with CBN for 48 h. The mRNA expression levels of the key melanogenic genes and their master transcription factors were analyzed. As shown in Figure 3, CBN treatment led to significant and dose-dependent downregulation of *Mitf*, *Tyr*, *Tyrp*1, and *Tyrp*2 mRNA levels compared to the untreated control. Specifically, at the highest concentration of 10 μM, the mRNA expression of *Mitf*, *Tyr*, *Tyrp*1, and *Tyrp*2 was suppressed by 18.0 ± 1.6%, 8.0 ± 0.5%, 18.3 ± 1.5%, and 5.9 ± 1.4%, respectively (Figure 3). These results confirm that CBN inhibits melanogenesis via transcriptional suppression of key melanin synthesis genes.

### 2.6. Immunoblotting Analysis of CBN’s Effects on Melanogenesis Signaling

**Figure 4:**
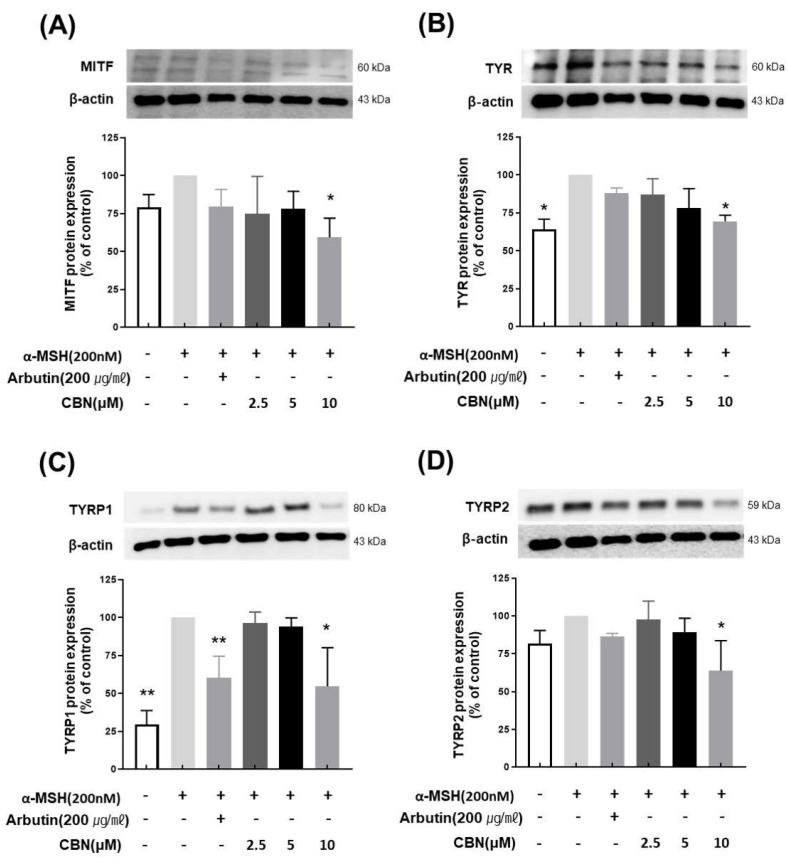
Cannabinol (CBN) downregulates the protein expression of key melanogenic factors. The anti-melanogenic effects of CBN were validated at the protein expression level by Western blot analysis. B16F10 melanoma cells were treated with various concentrations of CBN for 48 h. The Figure presents the protein bands (immunoblots) and corresponding quantitative graphs demonstrating that CBN treatment resulted in a dose-dependent downregulation of key melanogenic proteins and their master transcription factor: (**A**) MITF, (**B**) TYR, (**C**) TYRP1, and (**D**) TYRP2. GAPDH served as the loading control for normalization. The graphs show the relative protein expression levels, normalized to GAPDH and expressed as a percentage of the untreated control. *n* = 2 for α-MSH-untreated group, only α-MSH-treated group, and positive control; *n* = 4 for CBN-treated samples. All data are presented as the mean ± SD from independent experiments. * *p* < 0.05, ** *p* < 0.01.

**Figure 5:**
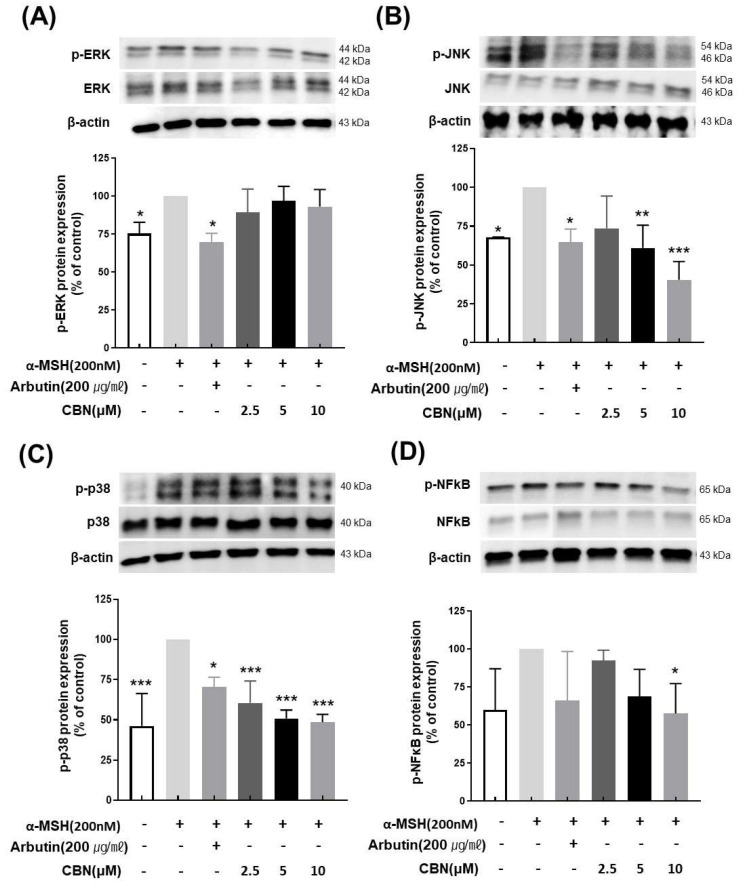
Cannabinol (CBN) modulates the phosphorylation of key proteins in the MAPK signaling pathway and the activation of NF-κB. The regulatory effect of CBN on upstream signaling was investigated by examining the mitogen-activated protein kinase (MAPK) pathway and the NF-κB transcription factor using Western blot analysis. B16F10 melanoma cells were treated with various concentrations of CBN for 48 h. The figure displays the immunoblots and corresponding quantitative graphs, which show the levels of total and phosphorylated proteins for the three major MAPK components and the expression of NF-κB: (**A**) ERK, (**B**) JNK, (**C**) p38 and (**D**) NF-κB. CBN treatment resulted in the modulation of the phosphorylated (p-) forms of these kinases, indicating the regulation of the MAPK signaling cascade, and the suppression of NF-κB protein level. GAPDH or the corresponding total protein served as the internal control for normalization. The graphs represent the relative phosphorylation levels, expressed as a ratio of the phosphorylated protein to the total protein, or as a percentage of the untreated control. *n* = 2 for α-MSH-untreated group, only α-MSH-treated group, and positive control; *n* = 4 for CBN-treated samples. All data are presented as the mean ± SD from independent experiments. * *p* < 0.05, ** *p* < 0.01, *** *p* < 0.001.

To evaluate the regulatory effects of CBN on melanogenesis at the protein level, we performed Western blot analysis on B16F10 melanoma cells treated with various concentrations of CBN (2.5, 5, and 10 μM) for 48 h. Consistent with our qRT-PCR findings, CBN treatment resulted in a dose-dependent reduction in the protein expression of key melanogenic markers, including MITF, TYR, TYRP1, and TYRP2. At the highest concentration of 10 μM, CBN markedly suppressed the expression of these proteins by 69.5 ± 3.9%, 54.7 ± 25.4%, 63.9 ± 19.8%, and 59.2 ± 12.7%, respectively, compared to untreated controls (Figure 4). Furthermore, we investigated the underlying upstream signaling pathways. As shown in Figure 5, CBN treatment led to a dose-dependent decrease in the protein phosphorylation of p-JNK and p-p38. Additionally, the protein levels of NF-κB, another key regulator of inflammation and melanogenesis, were significantly suppressed by CBN in a dose-dependent manner. Notably, while the graph suggested a slight increase in p-ERK phosphorylation following CBN treatment, this modulation was not confirmed as statistically significant in any group (Figure 5). These results collectively demonstrate that CBN exerts its potent anti-melanogenic effects primarily by selectively modulating the JNK and p38 MAPK pathways and the NF-κB pathway, while the ERK signaling pathway appears not to be a major target of CBN’s action in α-MSH-stimulated B16F10 cells. This selective modulation suggests a mechanism that maximizes anti-melanogenic outcomes by preserving the ERK pathway, which is often reported to play an inhibitory role in melanogenesis via MITF degradation.

## 3. Discussion

In the present study, we comprehensively investigated the anti-melanogenic effects of cannabinol (CBN) and elucidated the underlying molecular mechanisms in B16F10 melanoma cells. This study revealed for the first time that CBN acts as a potent inhibitor of melanogenesis and tyrosinase activity [4]. This effect was not due to cytotoxicity, thus confirming its potential as a safe and effective bioactive compound for skin-brightening applications [19]. Our data further revealed that CBN’s inhibitory action is mediated at the transcriptional level, as evidenced by the significant downregulation of key melanogenic genes, including MITF, TYR, TYRP1, and TYRP2 [20]. The coordinated downregulation of TYR, TYRP1, and TYRP2 enzymes, critical for both early and late stages of melanin biosynthesis, strongly suggests that CBN’s inhibitory effect is mediated by comprehensive transcriptional blockade rather than sole enzyme inhibition.

Most importantly, our results provide crucial mechanistic insights by showing that CBN suppresses melanogenesis through the selective modulation of upstream signaling pathways. Specifically, Western blot analysis demonstrated that CBN significantly and dose-dependently suppressed the phosphorylation of p38 and JNK, as well as the activation of NF-kB. However, it is important to note that CBN treatment did not affect the phosphorylation level of ERK. This finding is highly significant, as the MAPK cascade is a crucial regulator of MITF [21,22,23]. CBN’s ability to inhibit the pro-pigmentary p38 and JNK pathways while leaving the inhibitory ERK pathway intact suggests a highly optimized and potent depigmenting mechanism [24,25,26,27]. This p38/JNK selective regulation effectively removes the “accelerator” of melanogenesis while preserving the cell’s natural “brake” ERK, differentiating CBN from traditional broad-spectrum inhibitors such as kojic acid and arbutin, whose clinical use is often limited by adverse effects and a less comprehensive mechanism of action [28]. Furthermore, the concurrent suppression of p38 and JNK phosphorylation implies that CBN may act on a common upstream signaling regulator, such as a MAP2K (MKK3/6 or MKK4/7), or a receptor-mediated stress response that modulates the overall signaling balance governing melanogenic gene expression [29]. Such pathway-specific selectivity supports CBN’s non-toxic, physiological depigmenting effect, valuable for cosmetic and dermatological applications.

This observation is consistent with the hypothesis that CBN regulates MITF expression via multiple pathways. Melanin synthesis is predominantly activated by α-MSH signaling through the melanocortin 1 receptor (MC1R) to activate the cAMP/PKA/CREB cascade, which is a primary driver of MITF transcription [30,31,32]. Our data demonstrating the robust downregulation of MITF by CBN suggests that the compound may interfere with this crucial upstream cAMP/CREB signaling cascade in addition to its selective effects on the MAPK and NF-κB pathways [33,34]. In this regard, it is plausible that CBN indirectly modulates the MC1R-mediated cAMP signaling axis, potentially through receptor cross-talk or secondary messengers such as calcium or reactive oxygen species. These signaling intermediates are known to link cannabinoid receptor activation (CB1 and CB2) with intracellular MAPK and NF-κB modulation [35,36]. Thus, future investigations assessing receptor involvement using selective antagonists or CB1/CB2 knockout models could clarify whether CBN exerts its depigmenting action through canonical cannabinoid receptor signaling or through receptor-independent pathways.

**Figure 6:**
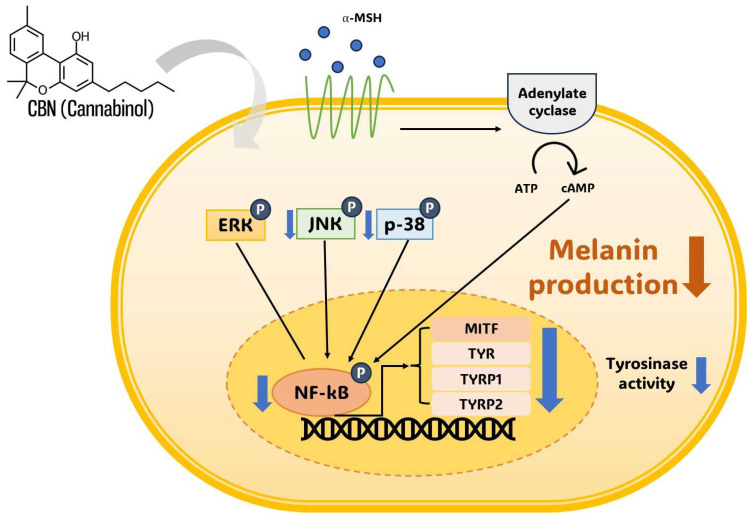
Schematic representation of cannabinol (CBN)-mediated anti-melanogenesis mechanism in B16F10 melanoma cells. Cannabinol (CBN) suppresses melanin production and tyrosinase activity by modulating key intracellular signaling pathways. Simultaneously, CBN modulates the MAPK pathway (JNK and p38) and suppresses the nuclear activity of the transcription factor NF-κB. The convergence of these inhibitory signals ultimately downregulates the expression of the master transcription factor MITF, which, in turn, decreases the expression of melanogenic enzymes, including TYR, TYRP1, and TYRP2. Arrows indicate signal flow (thin black) or CBN action (thick gray). ‘P’ in a blue circle denotes phosphorylation. Blue downward arrows indicate CBN-induced inhibition or downregulation of the target.

This proposed multi-target mechanism, combining the selective inhibition of p38 and JNK with the inhibition of NF-κB (and possibly cAMP/CREB), indicates that CBN may act as a powerful depigmenting agent by synergistically targeting MITF through multiple distinct signaling routes (Figure 6). At the downstream level, the robust suppression of MITF provides the molecular linkage for the observed coordinated reduction in its target genes TYR, TYRP1, and TYRP2. Given the established antioxidant properties of cannabinoids, this integrative regulation may encompass anti-inflammatory and redox-modulatory mechanisms that contribute to MITF-mediated pigment suppression, providing a clear mechanistic rationale for CBN’s multi-layered action on melanogenesis. Crucially, the simultaneous suppression of NF-kB is highly beneficial, as this transcription factor is a master regulator of inflammatory mediators that promote pigmentation following skin irritation or UV exposure, further expanding CBN’s utility beyond basic depigmentation [37,38].

In summary, our results demonstrated that CBN effectively suppressed key pro-melanogenic regulators, which is particularly significant. The selective suppression of the pro-pigmentary p38 and JNK provides a direct molecular link to the observed downregulation of MITF protein and mRNA. Furthermore, the inhibition of the NF-κB signaling pathway, a known mediator of inflammatory melanogenesis, offers another compelling mechanism for CBN’s anti-melanogenic effect [19,30]. This multifaceted modulation by CBN, selectively targeting both the pro-melanogenic MAPK components and the NF-κB cascade, suggests a robust and highly effective mechanism for its depigmenting action, which could be more efficacious than agents targeting a single pathway.

Nevertheless, it remains to be definitively elucidated whether CBN directly targets these signaling proteins or acts via upstream receptors or transcriptional regulators. To rigorously address this question, future studies employing genetic perturbation strategies, such as MITF or TYR knockdown/knockout (using siRNA or CRISPR/Cas9), will be essential. This methodology is critical for establishing causality, as previous work with the endocannabinoid anandamide (AEA) demonstrated that the stimulatory effect on melanogenesis was proven to be dependent on the presence of TYR and MITF after genetic knockdown [39]. Our data, showing a robust CBN-mediated suppression of these same master regulators, strongly suggest that CBN’s anti-melanogenic action will similarly prove to be MITF/TYR-dependent, underscoring the necessity of these follow-up genetic approaches. These techniques will definitively determine whether CBN’s effects rely on the integrity of these key melanogenic regulators or are mediated through broader signaling integration. Additionally, transcriptomic and phosphoproteomic analyses could help identify novel CBN-responsive targets and clarify whether its whitening effects are primarily receptor-driven, kinase-dependent, or redox-mediated. These comprehensive follow-up studies would decisively define the primary molecular targets of CBN and deepen the mechanistic understanding beyond correlation-level evidence.

The selective inhibition of melanogenesis by CBN, observed only in α-MSH-stimulated B16 cells and not under basal conditions, indicates that its mechanism of action primarily targets the α-MSH-dependent signal amplification cascade, rather than acting as a universal, constitutive suppressor of melanin synthesis. This selectivity is likely due to CBN downregulating the α-MSH–MC1R–cAMP–MITF pathway, effectively attenuating the stimulated increase in the expression of key melanogenic enzymes like tyrosinase. Crucially, the B16 cells maintain their basal tyrosinase activity and melanin synthesis because they rely on α-MSH-independent, constitutive pathways that are robust enough to sustain a physiological baseline and are not the primary target of CBN at the concentrations tested, thereby confirming that the compound is not cytotoxic but functions as a specific regulator of hyperpigmentation. This context-dependent modulation of signaling underscores CBN’s potential as a physiological “balancer” rather than a global inhibitor. Such precision is important for translational applications, as excessive inhibition of pigmentation can lead to hypopigmentation disorders. Therefore, CBN’s ability to normalize overactivated melanogenic signaling while preserving basal pigmentation supports its potential safety and applicability in long-term cosmetic or dermatological use. The successful synthesis and purification of CBN to over 99.0% purity using the iodine-mediated oxidative cyclization and CPC minimize the confounding effects of impurities, thereby strengthening the validity and reproducibility of our mechanistic conclusions [40]. Establishing this high-purity synthetic route is technically significant and provides a reliable basis for future pharmacological and clinical studies.

In conclusion, we successfully demonstrated the potent anti-melanogenic properties of high-purity CBN and defined its novel mechanism of action: the selective, multi-target suppression of pro-melanogenic p38 and JNK, alongside NF-kB inhibition, which collectively leads to MITF downregulation. This comprehensive, non-cytotoxic action posits CBN as a highly promising candidate for advanced functional cosmetics and dermatological applications. Future in vivo studies and omics-based analyses are warranted to definitively pinpoint the primary molecular targets and validate its clinical potential.

## 4. Materials and Methods

### 4.1. Plant Material and Preparation

The inflorescence of the hemp (*Cannabis sativa* ssp. *sativa*) cultivar, referred to as ‘Pink Pepper’ (GenBank accession number: GCA_029168945.11), was used in this study. This variety was recently developed by Lim [41] and is currently cultivated by the Chuncheon Bioindustry Foundation (CBF) in Chuncheon, Republic of Korea (37°53′33″ N, 127°44′38″ E). Freshly harvested inflorescences were first subjected to drying using a hot air dryer (Daedong, Daegu, Republic of Korea) at 40 °C for 48 h. Once fully dehydrated, the material was pulverized using a DA280-S grinder (Daesung, Paju, Gyeonggi-do, Republic of Korea) and passed through an 80-mesh sieve to achieve uniform particle size. The resulting powder was stored under controlled environmental conditions using a thermo-hygrostat (Model DH.DeADDBG1K, Daihan, Wonju, Gangwon-do, Republic of Korea), set to maintain a temperature of 23 °C and a relative humidity of 14%, until further use.

### 4.2. Conversion and Purification of CBN

CBD (10 g) extracted from *Cannabis sativa* L. was purified using the method described by Kim [42] and subsequently subjected to a conversion reaction to produce cannabinol (CBN). The reaction was performed in a mixture of iodine (15 g) and toluene (200 mL) under reflux conditions at 110 °C for 45 min with continuous stirring. After the reaction, the mixture was processed to separate the organic phase and prepared for purification. Centrifugal Partition Chromatography (CPC) was performed using a CPC-1000 system (Gilson, Middleton, WI, USA) to purify CBN to high purity. The separation employed a biphasic solvent system composed of n-hexane:ethyl acetate:methanol:water (5:5:5:5, *v*/*v*/*v*/*v*), operated in descending mode. In this system, the organic phase functioned as the stationary phase, whereas the aqueous phase served as the mobile phase. The CPC process was performed at a flow rate of 10 mL/min and a rotation speed of 1600 rpm. The collected fractions were analyzed by HPLC (Waters Corporation, Milford, MA, USA), and the CBN-rich fractions were pooled and concentrated. The highly purified CBN oil obtained after CPC purification was dissolved in 99% ethanol to prepare a 10 mM stock solution. This stock was subsequently diluted with the cell culture medium so that the final ethanol concentration did not exceed 0.1% (*v*/*v*) in any experiment.

### 4.3. Cell Culture and Viability Assay

The melanoma cell line B16F10 used in this study was obtained from ATCC (Rockville, MD, USA). Cells were cultured in Dulbecco’s Modified Eagle Medium (DMEM; GIBCO BRL, Grand Island, NY, USA) supplemented with 10% fetal bovine serum (FBS; GIBCO) and 1% penicillin/streptomycin (100 U/mL), and maintained in a humidified incubator at 37 °C with 5% CO_2_. The cells were passaged regularly to ensure optimal growth. Cell viability following CBN treatment was assessed using the Cell Counting Kit-8 (CCK-8; EZ-Cytox, DoGen, Seoul, Republic of Korea). B16F10 cells were seeded at a density of 2 × 10^4^ cells per well in 96-well tissue culture plates and treated with varying concentrations of CBN. Following a 48 h incubation period to allow for cell stabilization, CCK-8 reagent diluted 1:10 in phenol red-free DMEM was added to each well, and the plates were incubated for 1.5 h at 37 °C in a humidified incubator. The absorbance was measured at 450 nm using a spectrophotometric microplate reader (SpectraMax M5; Molecular Devices, San Jose, CA, USA). Cell viability was calculated relative to the absorbance of the wells containing the culture medium without cells.

### 4.4. Tyrosinase Inhibitory Activity Assay

Cells were seeded in a 96-well plate and incubated for 24 h. Subsequently, the cells were treated with α-melanocyte stimulating hormone (Melanogenesis stimulator; α-MSH; Sigma) and various concentrations of the test sample and further incubated for 48 h. After the incubation period, the cells were washed twice with cold phosphate-buffered saline (PBS; GIBCO). In addition, 250 µL of Lysis buffer (specifically formulated for depigmentation assays) was added to each well, and the plate was stored in a deep freezer for a minimum of 1 h to ensure complete cell lysis. The plate was removed from the deep freezer, thawed on ice for 30 min, and the cell lysate was harvested using a cell scraper. The resulting lysate was centrifuged at 12,000 rpm for 15 min, and the supernatant containing the enzyme was collected. For the tyrosinase activity assay, 60 µL of the collected supernatant was mixed with 140 µL of L-DOPA (L-3,4-dihydroxyphenylalanine, 2 mg/mL in a new 96-well plate. The mixture was reacted at 37 °C for 1 h. The absorbance was then measured at 490 nm using a microplate reader. Each experiment was performed independently in triplicate. The relative tyrosinase activity was calculated by comparing the absorbance of the treated sample groups to the control group (treated only with α-MSH and vehicle). Arbutin (Sigma, St. Louis, MO, USA) at 100 µg/mL was used as a positive control.

### 4.5. Measurement of Melanin Content in B16F10 Cells

The melanin content assay was performed based on the method described by Hosoi [43] with slight modifications. B16F10 cells were seeded at a density of 1 × 10^5^ cells per 60 mm dish and cultured for 24 h. Cells were then treated with 200 nM α-MSH along with varying concentrations of CBN. After 48 h of treatment, the culture medium was removed and the cells were washed three times with PBS. Melanin was solubilized by incubating the cells with 100 µL of 1 N NaOH (Sigma) at 60 °C for 2 h. The absorbance of solubilized melanin was measured at 405 nm using a spectrophotometric microplate reader. Arbutin (100 µg/mL; Sigma-Aldrich) was used as positive control. Melanin inhibition was expressed as the percentage of melanin content in cells treated with α-MSH alone. All experiments were independently repeated thrice.

### 4.6. RNA Extraction and Real-Time RT-PCR Analysis

Total RNA was isolated using the PureLinkTM RNA Mini Kit (Invitrogen) according to the manufacturer’s instructions. Complementary DNA (cDNA) was synthesized from 1 µg of extracted RNA using 10 µL of high-capacity cDNA reverse transcription kit (Applied Biosystems, Foster City, CA, USA). To evaluate the expression of specific markers in B16F10 cells, real-time reverse transcription PCR (RT-PCR) was performed using a Quant Studio™ 5 System (Applied Biosystems, Foster City, CA, USA). The primers used for the analysis were: *Tyr* (Forward: 5′-CTCTGGGCTTAGCAGTAGGC-3’, Reverse: 5′-GCAAGCTGTGGTAGTCGTCT-3’), *Tyrp*1 (Forward: 5′-CCCCTAGCCTATATCTCCCTTTT-3’, Reverse: 5′-TACCATCGTGGGGATAATGGC-3’), *Tyrp*2 (Forward: 5′-TTCTGCTGGGTTGTCTGGG-3’, Reverse: 5′-CACAGATGTTGGTTGCCTCG-3’), *Mitf* (Forward: 5′-ACTTTCCCTTATCCCATCCACC-3’, Reverse: 5′-TGAGATCCAGAGTTGTCGTACA-3’) and *GAPDH* (Forward: 5′-AGGTCGGTGTGAACGGATTTG-3’, Reverse: 5′-TGTAGACCATGTAGTTGAGGTCA-3’). The qPCR was conducted under the following cycling conditions: initial denaturation at 95 °C for 5 min, followed by 40 cycles of denaturation at 95 °C for 15 s, annealing at 58 °C for 15 s, and extension at 72 °C for 30 s. Gene expression levels were quantified relative to GAPDH as the internal control using the comparative Ct (ΔΔCt) method.

### 4.7. Immunoblotting Analysis

B16F10 cells were seeded at a density of 2.5 × 10^5^ cells per well in 6-well plates and incubated for 24 h. The cells were then treated with CBN at concentrations of 2.5, 5, and 10 μM, in combination with 200 nM α-MSH, for an additional 48 h. Total protein was extracted using PRO-PREP™ (iNtRON Biotechnology, Seongnam, Gyeonggi-do, Republic of Korea) and quantified with the BCA Protein Assay Kit (Thermo Fisher Scientific, Rockford, IL, USA). Proteins were separated by sodium dodecyl sulfate-polyacrylamide gel electrophoresis and transferred to membranes, which were then blocked with 5% bovine serum albumin (BSA). All primary antibodies, TYR, TYRP1, TYRP2, MITF, ERK, JNK, p38, NF-κB, and β-actin were purchased from Cell Signaling (Danvers, MA, USA). Target proteins were visualized using Super Signal Western Blot Enhancer (Pierce, Waltham, MA, USA), and signal detection was performed using the LAS-4000 imaging system (Fujifilm, Tokyo, Japan). Each condition was analyzed in independent biological replicates as follows: α-MSH-untreated group, only α-MSH-treated group, and positive control (*n* = 2 each); CBN 2.5 μM, 5 μM, and 10 μM (*n* = 4 each). Densitometric values were obtained from independent biological replicates and normalized to β-actin.

### 4.8. Data Analysis

All experiments were performed independently at least three times, and the results are presented as the mean ± standard deviation (SD). Different letters above the bars indicate significant differences (*p* < 0.05). Data was visualized using GraphPad Prism software (version 8.0.1, GraphPad Software). Statistical significance among groups was evaluated using one-way analysis of variance (ANOVA), validated at significance levels of 5%, 1%, and 0.1% (* *p* < 0.05, ** *p* < 0.01, *** *p* < 0.001).

## 5. Conclusions

In conclusion, our study successfully demonstrated that cannabinol (CBN) possesses potent anti-melanogenic properties without inducing cytotoxicity. We found that CBN exerts its inhibitory effects by downregulating the expression of key melanogenic genes and proteins, including MITF, TYR, TYRP1, and TYRP2. Our most significant finding was that this action was mediated through the selective suppression of crucial upstream signaling pathways: specifically, the p38 and JNK components of the MAPK cascade and the NF-κB pathway. This selective modulation targeting pro-melanogenic pathways, while preserving the ERK pathway, provides a comprehensive explanation for CBN’s powerful skin-brightening effects, positioning it as a promising new bioactive compound for cosmetic and therapeutic applications in hyperpigmentation. Further in vivo and clinical studies are warranted to validate these findings and explore their full potential.

## Figures and Tables

**Table 1:** Centrifugal Partition Chromatography (CPC) Conditions.

Time (min)	Flow Rate (mL/min)	Rotor Speed (rpm)	Solvent A (%)	Solvent B (%)	Solvent C (%)
Equilibration					
0.0–7.0	300	500	2	77	21
7.0–12.0	200	2000	100	0	0
Elution					
0.0–20.0	200	1500	100	0	0
20.0–30.0	200	1500	2	77	21

The solvent system consisted of (A) Hexane, (B) Methanol, and (C) Deionized Water. The pump injection was set at a flow rate of 30 mL/min with an injection volume of 30 mL. Detection was performed at 220, 263, and 280 nm, with a scan range from 200 to 400 nm. A total volume of 300 mL was collected.

## Data Availability

Data is contained within the article. Materials of the CBN isolated from ‘Pink Pepper’ provide from the corresponding authors.
